# An Analysis of the *Athetis lepigone* Transcriptome from Four Developmental Stages

**DOI:** 10.1371/journal.pone.0073911

**Published:** 2013-09-13

**Authors:** Li-Tao Li, Yan-Bin Zhu, Ji-Fang Ma, Zhi-Yong Li, Zhi-Ping Dong

**Affiliations:** 1 Institute of Millet Crops, Hebei Academy of Agriculture and Forestry Sciences, Shijiazhuang, China; 2 College of Life Science, Agricultural University of Hebei, Baoding, China; Kyushu Institute of Technology, Japan

## Abstract

*Athetis lepigone* Möschler (Lepidoptera: Noctuidae) has recently become an important insect pest of maize (*Zea mays*) crops in China. In order to understand the characteristics of the different developmental stages of this pest, we used Illumina short-read sequences to perform *de novo* transcriptome assembly and gene expression analysis for egg, larva, pupa and adult developmental stages. We obtained 10.08 Gb of raw data from Illumina sequencing and recovered 81,356 unigenes longer than 100 bp through a de novo assembly. The total sequence length reached 49.75 Mb with 858 bp of N50 and an average unigene length of 612 bp. Annotation analysis of predicted proteins indicate that 33,736 unigenes (41.47% of total unigenes) are matches to genes in the Genbank Nr database. The unigene sequences were subjected to GO, COG and KEGG functional classification. A large number of differentially expressed genes were recovered by pairwise comparison of the four developmental stages. The most dramatic differences in gene expression were found in the transitions from one stage to another stage. Some of these differentially expressed genes are related to cuticle and wing formation as well as the growth and development. We identified more than 2,500 microsatellite markers that may be used for population studies of *A.* *lepigone*. This study lays the foundation for further research on population genetics and gene function analysis in *A*. *lepigone*.

## Introduction

*Athetis lepigone* Möschler (Lepidoptera: Noctuidae) is found in many countries in Europe and Asia [1]–[5]. In 2005 it was first discovered that *A.* *lepigone* causes severe damage to maize crops in China [6], although the species had never before been documented as an agricultural pest. In subsequent years the range of occurrence has expanded and increasing crop damage has provoked interest in *A.* *lepigone*. In 2011, an *A.* *lepigone* outbreak occurred in Shandong, Shanxi, Henan, Anhui and Jiangsu provinces, over an area about 2.2 million ha. *A.* *lepigone* is now considered an important pest of maize in China.

In the Huang-Huai region of China *A.* *lepigone* can produce up to four generations a year and the larvae of the second generation cause the majority of the damage to the maize crop [7]. In maize fields larvae cluster together in straw next to maize seedlings and damage the seedlings in several different ways. Larvae drill into and eat young maize stems, resulting in wilt and death of the seedling. They also eat maize prop root, causing lodging and severe yield loss of maize crops [8].

*A.* *lepigone* is a generalist herbivore and has been observed eating 30 species of plants from 13 different plant families [8]. *A.* *lepigone* overwinters in the north of China, where cocooned instar larva can supercool to withstand temperatures reaching −25°C [9]. Both male and female adults can mate multiple times and females may produce up to 500 offspring at a time [10]. This species was previously known to exist in the region, but populations seem to have grown rapidly in recent years. *A.* *lepigone* populations may be benefitted by agricultural practices that return a large amount of straw to fields after the wheat harvest that is then planted with maize. This creates a suitable habitat and rich food source for *A.* *lepigone*. The availability of straw shelter appears to be the key factor influencing the outbreak of *A.* *lepigone*, rather than migration or diffusion from other regions [11].

As a newly discovered pest, very few genetic resources are available for *A.* *lepigone*. At publication time, there were only 17 COI haploid gene sequences of *A.* *lepigone* in the NCBI database, yet genetic information is urgently needed to study the molecular regulation mechanisms of *A.* *lepigone* and better understand how to control *A.* *lepigone* damage to maize crops.

In recent years, next-generation high-throughput DNA sequencing techniques have provided fascinating opportunities in the life sciences and dramatically improved the efficiency and cost-effectiveness of gene discovery [12]. Next-gen sequencing generates a large amount of data and for that reason has been widely used in the study of humans [13], crops [14], [15], agricultural pests [16] and other model organisms [17], [18]. For example, Xue et al. sequenced the transcriptome of different development stages of the brown planthopper (BPH) *Nilaparvata lugens*, obtaining 85,526 unigenes, and found a large number of genes related to wing dimorphism and sex difference [19]. In another landmark study transcriptome sequencing was performed on 30 distinct developmental stages of *Drosophila melanogaster* [18]. This study identified 111,195 new functional elements, including coding and non-coding genes and splicing and editing events. In 2012 Chen et al. contrasted the transcriptomes of full-sister queen-larvae (QL) and worker-destined larvae (WL) using high-throughput RNA-Seq [20]. They found more than 4,500 genes with different expression between the two types larvae, of which more than 70% were up-regulated in QL. They also confirmed that a higher juvenile hormone titer was necessary for QL development.

In this study we sequenced and analyzed the transcriptome of *A.* *lepigone* to understand differences in gene expression in different developmental stages and create a basis of molecular information for the development of molecular markers, identification of genes and the functional analysis of expressed genes.

## Materials and Methods

### Ethics Statement

*Athetis lepigone* Möschler (Lepidoptera: Noctuidae) is not an endangered or protected species and has recently become an important insect pest of maize (*Zea mays*) crops in China. A recent, serious outbreak of *A.* *lepigone* in Shijiazhuang, Hebei Province, provided ample opportunity for sample collection. There were no specific permits which were required for these locations/activities for collection.

### Source of *A.* *lepigone* Material

The *A.* *lepigone* strain was collected from maize fields in Shijiazhuang, Hebei Province, China in July 2011 and kept indoors at a temperature of 26±1°C with a 14∶10 h light:dark photoperiod for eight generations. Samples used in this study were collected at four developmental stages of the ninth generation: 1) a mixture of three-day-old eggs; 2) a mixture of fifth instar larvae; 3) a mixture of three-day-old pupae and 4) a mixture of emergent male and female adults. Every sample was immediately frozen in liquid nitrogen and stored at −80°C until extraction.

### RNA Isolation, cDNA Library Preparation and Illumina Sequencing for Transcriptome Analysis

Total RNA was extracted using the TRIzol Reagent (Invitrogen) according to the manufacturer’s protocol. The mRNA was purified from total RNA using oligo (dT) magnetic beads and fragmented into small pieces in fragmentation buffer at 70°C for 4 min. First strand cDNA synthesis used SuperScript II and random primers and second strand cDNA synthesis used DNA polymerase I and RNaseH. The double-stranded cDNA was end-repaired using T4 DNA polymerase, Klenow Enzyme (NEB), and T4 polynucleotide kinase (NEB) followed by a single A base addition using Klenow exo-polymerase, then ligated with Adapter using DNA ligase (NEB). The products of the ligation reaction were amplified by PCR and purified using the QIAquick PCR Purification Kit (Qiagen) to create a cDNA library. The cDNA library was quantified with Qubit (Invitrogen) and a cluster of the DNA fragment was amplified using bridge PCR on the surface of a flow cell chip. When the single molecular DNA cluster was amplified many times, the products were sequenced on an Illumina GAII.

### Assembly and Gene Identification

The reads obtained for each sample were assembled separately using Trinity software (http://trinityrnaseq.sourceforge.net/) with the K-mer = 25 [21]. Contig were obtained by extending based on the overlap between sequences. Next, the resultant contigs were joined into transcripts with the paired-end information. We selected the longest transcript from the potential alternative splicing transcripts as the sample unigene. The unigenes from four samples were combined to create a lifetime unigene database of *A.* *lepigone* by the BLAST-Like Alignment Tool (http://genome.ucsc.edu/cgi-bin/hgBlat). All raw transcriptome data have been deposited in the NIH Short Read Archive (SRA) with the accession number SRP019964.

### Functional Annotation for Unigenes

Unigenes were aligned with the Nr, Nt, Swissprot, TrEMBL databases using BLAST with a cut-off e-value of 10^−5^. Blast2GO was used to obtain Gene ontology (GO) annotation of the unigenes. The COG and KEGG pathway annotation were also performed using BLAST against Cluster of Orthologous Groups databases and Kyoto Encyclopedia of Genes and Genomes. All above searching were performed with a cut-off e-value of 10^−5^.

### Microsatellite Identification

Unigenes ≥1 Kb were subjected to SSR analysis with the Microsatellite Identification tool (MISA; http://pgrc.ipk-gatersleben.de/misa/). Detection criteria were constrained to perfect repeat motifs of 1–6 bp and a minimum repeat number of 10, 6, 5, 5, 5 and 5, for mono-, di-, tri-, tetra-, penta- and hexa-nucleotide microsatellites, respectively.

### Differential Gene Expression Analyses

In each sample the expression abundance of unigenes was obtained by mapping the reads from four samples against the reference set of *A.* *lepigone* lifetime unigenes using Blat software. The relative transcript abundance were output as RPKM values (Reads Per Kilobase of exon model per Million mapped reads) [22], which is calculated as
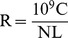



Where C is the number of mappable reads that fell onto the specific unigene, N is the total number of reads that could map to unigenes in that sample, and L is the length of the unigene. Differentially expressed genes were detected by IDEG6 software (http://telethon.bio.unipd.it/bioinfo/IDEG6/) with a general chi square test based on RPKM values. The test result was corrected by FDR (false discovery rate). Genes were regarded as differentially expressed when the FDR<0.01 and the absolute value of the log2 ratio >1 (the RPKM values of the gene in one sample was at least 2 times that of the gene in another sample).

## Results

### Illumina Sequence Data and Assembly

The cDNA samples extracted from the eggs (SRX254872), larvae (SRX254873), pupae (SRX254874) and adults (SRX254875) of *A.* *lepigone* were sequenced using the Illumina sequencing platform and each sample generated more than 2 G of transcriptome data. The larvae sample produced the least amount of data (2265.42 Mb) and the pupae sample produced the most data (2857.80 Mb). The average quality value was ≥20 for more than 99.5% of the cycle (Table 1), suggesting the sequencing was highly accurate. Sample GC content was consistently about 48%. The number and total length of the transcripts and unigenes were different for each sample, but the distribution of lengths was consistent (Table 2). Assembled reads from the four samples resulted in 81,356 unigenes with a total length of 49.75 Mb, a mean length of 611.5 bp and an N50 length of 858 bp. Out of these 81,356 unigenes, 12,070 unigenes were ≥1000 bp, accounting for 14.84% of the total.

**Table 1:** Summary for raw reads of four samples.

Sample	Data (Mb)	GC%^a^	CycleQ 20%^b^
Egg	2608.97	48.12	99.5
Larva	2265.42	48.09	99.5
Pupa	2857.8	45.99	100
Adult	2597.59	46.99	99.5

Note:

a GC base content.

b The proportion of cycle, of which the average quality value is ≥20.

**Table 2:** Summary for *A.* *lepigone* transcriptome.

Gene length	Egg		Larva		Pupa		Adult		*A.lepigone*
	Transcriptnumber	Unigenenumber	Transcriptnumber	Unigenenumber	Transcriptnumber	Unigenenumber	Transcriptnumber	Unigenenumber	Unigenenumber
200–300	23619	12537	16052	10967	20584	16022	18808	11558	28165
300–500	25624	12825	17798	11841	19209	13520	21826	12820	25257
500–1000	22622	9624	13885	7738	16843	10179	19196	9105	15864
1000–2000	13260	5562	6859	3486	9311	4994	10581	4877	8556
2000+	5515	2166	1673	765	3642	1655	3407	1409	3514
Total Length	66340657	28834461	34397106	19348688	47956499	27815088	50511500	24683128	49749348
Count	90640	42714	56267	34797	69589	46370	73818	39769	81356
N50_Length	1064	971	806	689	986	805	943	838	858
Mean Length	731.91	675.06	611.32	556.04	689.14	599.85	684.27	620.66	611.50

### Annotation of Predicted Proteins

**Figure 1:**
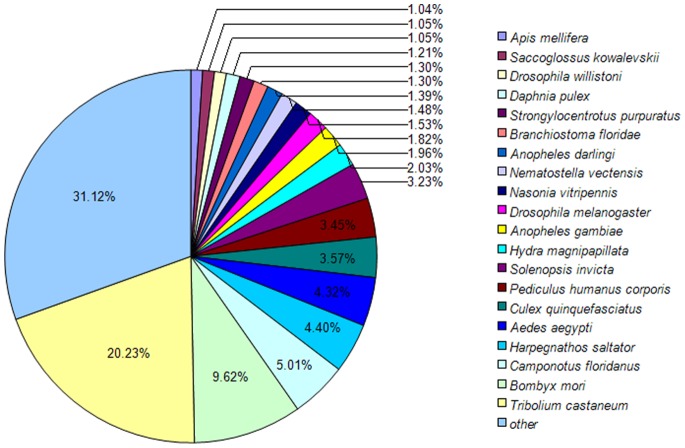
Species distribution of the BLASTX results. Note: This figure shows the species distribution of unigene BLASTX results against the NCBI-Nr protein database with a cutoff E value <10^−5^. Different colors represent different species. Species with proportions of more than 1% are shown.

We used BLASTX to query various protein databases and annotate 81,356 unigene sequences. 33,736 unigenes (41.47% of the *A.* *lepigone* unigenes) had significant matches in the Nr database, 17,762 (21.83%) had significant matches in the Nt database, 27,909 (34.30%) had significant matches in the Swissprot database, 37754 (46.41%) unigenes had similarity to proteins in the TrEMBL database. Altogether, 43,189 (53.09%) unigenes were successfully annotated (Table 3). Nr database queries revealed that a high percentage of *A.* *lepigone* sequences closely matched sequences of the red flour beetle (*Tribolium castaneum*, 20.23%), silkworm (*Bombyx mori*, 9.62%), Florida carpenter ant (*Camponotus floridanus*, 5.01%), India jumping ant (*Harpegnathos saltator*, 4.40%) and the yellow fever mosquito (*Aedes aegypti*, 4.32%, Figure 1). There were 20 species in the Nr database with which *A.* *lepigone* had >1% match (Table S1).

**Table 3:** Functional annotation of *A.* *lepigone* unigenes.

Annotated database	Annotated Number	300≥length<1000	length≥1000
Nr Annotation	33736	17425	9829
Nt Annotation	17762	7894	7133
Swissprot Annotation	27909	13661	8847
TrEMBL Annotation	37754	19769	10121
COG Annotation	11518	5210	3792
GO Annotation	15585	7446	5412
KEGG Annotation	11824	5557	3573
All Annotated	43189	22409	10522

### SSR Development and Analysis

By using MISA, we identified 2,819 simple sequence repeats (SSRs) or microsatellites, distributed in 2,411 sequences, and accounting for 2.96% of the total number of unigenes. Trinucleotide repeats are the most common form of microsatellite present (13.66%), followed by dinucleotide repeats (6.63%) and compound nucleotide repeats (3.83%; Table 4). Among the trinucleotide repeats, the CGC/GCC/GAA/GGC/AAT/AAG/CAA/TGA types are the most common repeats in the recovered unigenes (39.48%). GC/TG/AT/TA repeats account for 54.9% of the total number of repeats in the unigenes and the (GC)_n_ motif is the most frequently recovered dinucleotide repeat.

**Table 4:** Summary of simple sequence repeat (SSR) types in the *A.* *lepigone* transcriptome.

SSR type	Repeat motif	Numbers	All/frequency
Mono-nucleotide	(A) n/(T) n/(C) n/(G) n	1383/779/43/32	2123/75.31%
Di-nucleotide	GC/TG/AT/TA/CA/CG/AC/GT/AG/TC/CT/GA	33/23/23/22/17/16/14/13/9/9/8/8	184/6.53%
Tri-nucleotide	CGC/GCC/GAA/GGC/AAT/AAG/CAA/TGA/ATA/ATT/AGA/TAT/GCG/TTC/CCG/CAC/TCA/GAT/ACA/CGG/CCA/ACC/TTA/TTG/TCT/GAG/CTT/ATG/CAG/TAA/GAC/CAT/CGA/AAC/ATC/AGC/GGT/GCA/GCT/CTG/AGT/ACG/TGT/TGC/TCG/GTT/GTG/CTC/AGG/ACT/TAC/TGG/TCC/GTC/GGA/CTA/CGT/CCT	22/22/20/19/19/18/17/15/14/14/11/11/11/9/9/8/8/8/7/7/7/6/6/6/6/6/6/5/5/4/4/4/4/3/3/3/3/3/3/3/2/2/2/2/2/2/2/2/1/1/1/1/1/1/1/1/1/1	385/13.66%
Tetra-nucleotide	ATTT/AATT/GTAC/TGCG/ACTC/AAGA/AAAT/ATTC/TGAT/TGTA/TGTT/TTAT/TTAG	3/1/1/1/1/1/1/1/1/1/1/1/1	15/0.53%
Penta-nucleotide	TGGTA/TATTC/GAGAA/TCAAA	1/1/1/1	4/0.14%
Compound SSR			108/3.83%
Total			2819

### Gene Ontology (GO), Clusters of Orthologous Groups (COG) and Kyoto Encyclopedia of Genes and Genomes (KEGG) Ontology (KO) Classification

**Figure 2:**
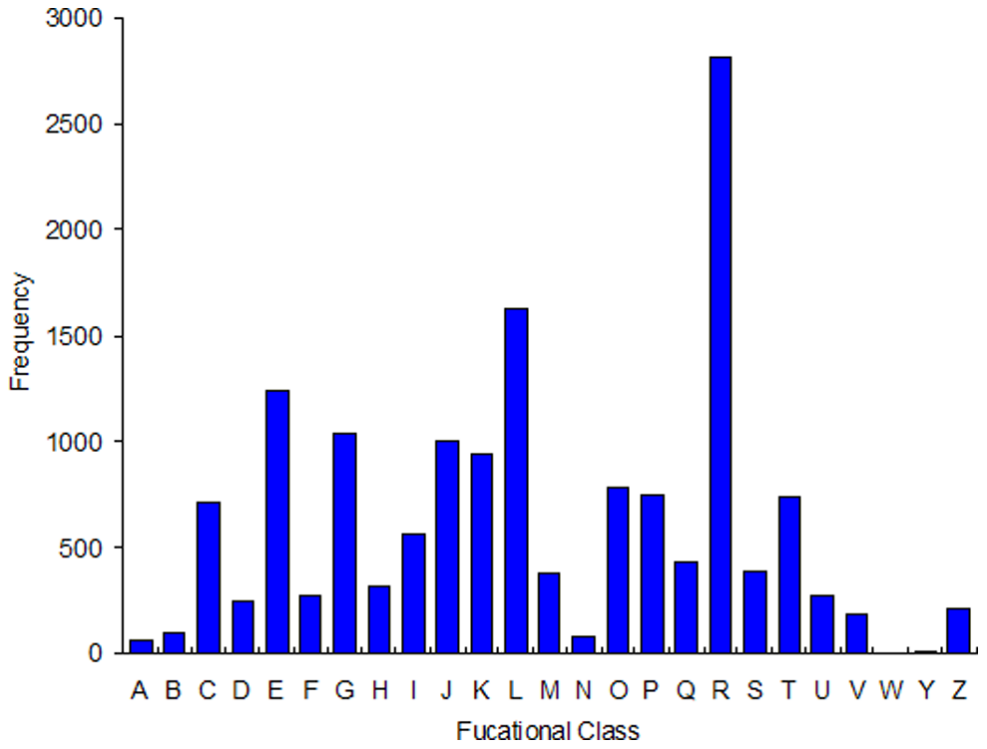
COG categories of the unigenes. Note: Class definition, Number of this class, Percent of this class (%). A: RNA processing and modification, 60, 0.40. B: Chromatin structure and dynamics, 100, 0.66. C: Energy production and conversion, 717, 4.74. D: Cell cycle control, cell division, chromosome partitioning, 244, 1.61. E: Amino acid transport and metabolism, 1234, 8.16. F: Nucleotide transport and metabolism, 273, 1.81. G: Carbohydrate transport and metabolism, 1032, 6.83. H: Coenzyme transport and metabolism, 312, 2.06. I: Lipid transport and metabolism, 560, 3.70. J: Translation, ribosomal structure and biogenesis, 994, 6.58. K: Transcription, 938, 6.20. L: Replication, recombination and repair, 1632, 10.80. M: Cell wall/memberance/envelope biogenesis, 378, 2.50. N: Cell motility, 78, 0.52. O: Posttranslational modification, protein turnover, chaperones, 778, 5.15. P: Inorganic ion transport and metabolism, 746, 4.93. Q: Secondary metabolites biosynthesis, transport and catabolism, 432, 2.86. R: General function prediction only, 2811, 18.59. S: Function unknown, 384, 2.54. T: Signal transduction mechanisms, 736, 4.87. U: Intracellular trafficking, secretion, and vesicular transport, 273, 1.81. V: Defense mechanisms, 186, 1.23. W: Extracellular structures, 0, 0. Y: Nuclear structure, 6, 0.04. Z: Cytoskeleton, 212, 1.40.

We classified the functions of the predicted *A.* *lepigone* unigenes by GO, COG and KO analysis. In the COG functional classification, 11,518 unigenes could be annotated by 15,117 functions involved in 24 COG categories (Figure 2). Among them, the general function prediction was the largest group (2,811 genes, 18.59%), followed by the functions of replication, recombination and repair (1632, 10.80%), amino acid transport and metabolism (1234, 8.16%), carbohydrate transport and metabolism (1032, 6.83%), translation, ribosomal structure and biogenesis (994, 6.58%) and transcription (938, 6.20%). Interestingly, we did not find any genes related to “extracellular structures”. Genes annotated as “RNA processing and modification” (60, 0.40%), “chromatin structure and dynamics” (100, 0.66%), “cell motility” (78, 0.52%) and “nuclear structure” (6, 0.04%) represent the smallest groups predicted by COG.

**Figure 3:**
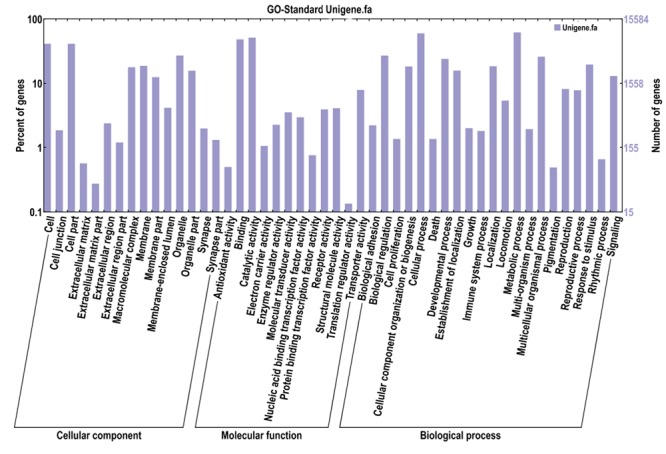
GO categories of the unigenes. Note: The unigenes are annotated in three main categories: biological process, cellular component and molecular function. The right y-axis indicates the number of genes in a category. The left y-axis indicates the percentage of a specific category of genes in that main category.

15,585 unigenes were annotated with 98,773 GO functions based on sequence similarity, with an average of 6.34 GO annotations per unigene (Table S2). The three main categories of GO annotations were 29,384 GO annotations (29.75%) for cellular component, 19,838 annotations (20.08%) for molecular function and 49,550 annotations (50.17%) for biological process. The main categories can be subdivided into 60 categories (Figure 3, Table 5). In each of the three main categories, “cell”, “catalytic activity” and “metabolic process” categories were the highest proportion of annotations, followed closely by “cell part”, “binding” and “cellular process”. In addition, there were 12 function subclasses with only a few genes (Table 5).

**Table 5:** GO categories contain few genes.

Function class		Number of genes	Percent of genes(%)
Cellular component	nucleoid	1	0.006
	virion	2	0.013
	virion part	2	0.013
Molecular function	channel regulator activity	6	0.038
	metallochaperone activity	6	0.038
	morphogen activity	6	0.038
	nutrient reservoir activity	2	0.013
	protein tag	2	0.013
	receptor regulator activity	3	0.019
Biological process	carbon utilization	2	0.013
	cell killing	4	0.026
	viral reproduction	1	0.006

To identify the biological pathways that are active in *A.* *lepigone*, we mapped the unigene sequences to the reference canonical pathways in the Kyoto Encyclopedia of Genes and Genomes (KEGG). In total, we assigned 11,824 sequences to 280 KEGG pathways (Table S3).

### Differential Gene Expression at Four Developmental Stages

**Figure 4:**
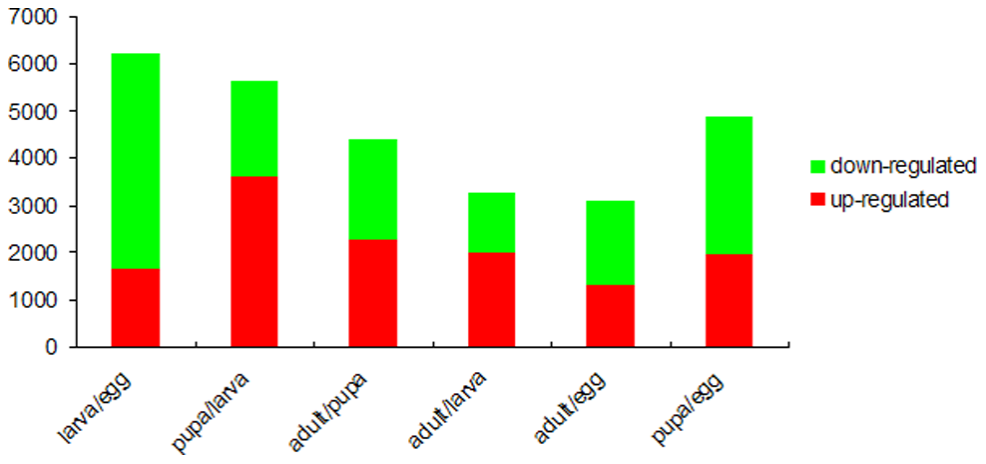
Numbers of DGE unigenes in each comparison.

Differentially expressed genes at four developmental stages were identified in IDEG6. Significantly different expression levels were found in 6,202 genes in the egg and larva libraries (Figure 4). Among those genes, 1,620 were up-regulated in the larva stage and 556 genes were uniquely expressed in larva. 4,582 genes were down-regulated in the larva stage and 1,490 genes were uniquely expressed in the egg. The twenty genes exhibiting the strongest up-regulated and down-regulated genes in the larva and egg unigene comparison are shown in Table S4. All ten genes up-regulated in the larvae stage have predicted functions, including three cuticular protein genes (*putative cuticle protein*, *cuticular protein CPR2* and *putative cuticle protein CPG36*). Seven of the ten down-regulated genes have predicted functions, *i*.*e*., three cuticular protein genes (*cuticular protein CPG8*, *cuticular protein RR-2 motif 101* and *cuticular protein CPG7*), a serine protease inhibitor gene (*serine protease inhibitor 8*) and a chondroitin gene (*Chondroitin proteoglycan-2*). According to the COG classification (Table S5), the genes that exhibit up-regulated expression in the larva stage mainly correlate to energy, material transport and metabolism, *i*.*e*., energy production and conversion, carbohydrate transport and metabolism, amino acid transport and metabolism, lipid transport and metabolism, inorganic ion transport and metabolism, secondary metabolites biosynthesis, transport and catabolism, and all genes related to RNA processing and modification, intracellular trafficking, secretion, and vesicular transport and chromatin structure and dynamics, were down-regulated in larva.

In total, 5,609 genes demonstrate significant changes between the pupa and larva libraries (Figure 4). In the pupa library, 3,616 genes were up-regulated and 1,993 genes were down-regulated. 966 genes were uniquely expressed in the pupa while 754 genes were uniquely expressed in the larva. Nine of the ten most up-regulated genes in the pupa library have predicted functions (Table S4), *i*.*e*., an apolipoprotein gene (*32 kDa apolipoprotein*), a neuropeptide related gene (*neuropeptide-like precursor 4*), a cuticular protein gene (*cuticular protein hypothetical 12*) and an enzyme gene (*similar to Chymotrypsin-2*). All ten of the most down-regulated genes have predicted functions, *i*.*e*., five cuticular protein genes (*putative cuticle protein CPG36*, *Larval cuticle protein 16/17*, *cuticular protein CPR2*, *Larval cuticle protein 1* and *putative cuticular protein*) and three enzyme genes (*serine protease 28*, *lipase* and *trypsin T2a*). Based on the COG functional classification (Table S5), up-regulated genes in the pupa were involved in RNA processing and modification, transcription, replication, recombination and repair, chromatin structure and dynamics, cell cycle control, cell division, chromosome partitioning, signal transduction mechanisms and cytoskeleton.

A comparative analysis between the adult and pupa libraries revealed 4,399 genes with significant expression changes (Figure 4). Among these genes, 2,273 were up-regulated in the adult stage and 2,126 were down-regulated in the adult. 725 genes were uniquely expressed in the adult stage and 733 genes were uniquely expressed in the pupa. Nine of the ten most up-regulated genes in the adult library have predicted functions (Table S4), *i*.*e*., three yolk formation related genes (*yolk polypeptide 2* and 2 *vitellogenin*), two flying related genes (*troponin C type IIIa-like protein* and *flightin*) and one antimicrobial peptide gene (*Anionic antimicrobial peptide 2*). Nine of the ten most down-regulated genes have predicted functions, *i*.*e*., an energy metabolism related gene (*NADH dehydrogenase subunit 2*) and two amino acid-rich protein genes (*Cysteine and glycine-rich protein 1* and *glycine/tyrosine-rich eggshell protein*). In the COG classification (Table S5), the up-regulated genes in the adult stage are involved in translation, ribosomal structure and biogenesis, defense mechanisms, intracellular trafficking, secretion and vesicular transport and energy production and conversion.

There were 3,254 genes with demonstrated significant changes between the adult and larva libraries (Figure 4). In the adult library 1,979 genes were up-regulated and 1,275 genes were down-regulated. 576 genes were uniquely expressed in the adult and 505 genes were uniquely expressed in the larvae. The eight most up-regulated genes in the adult library have the following predicted functions (Table S4), *i*.*e*., a cuticular protein gene (*AF117586_1 putative cuticle protein*), an egg formation gene (*yolk polypeptide 2*), a signal perception gene (*chemosensory protein*) and two enzyme genes (*similar to cathepsin F like protease* and *beta-fructofuranosidase*). Nine of the most down-regulated genes were annotated as an intestinal mucin gene (*intestinal mucin SeM8*), a molting hormone regulated protein (*ecdysteroid-regulated protein*), two cuticular protein genes (*putative cuticle protein CPH37* and *Larval cuticle protein 16/17*) and two serine protease genes (*serine protease 11 and serine protease 28*). According to the COG classification (Table S5), up-regulated genes in the adult stage compared to larvae were mainly involved in translation, ribosomal structure and biogenesis, transcription, replication, recombination and repair, cell cycle control, cell division, chromosome partitioning, signal transduction mechanisms, intracellular trafficking, secretion and vesicular transport.

Comparison of the adult and egg libraries revealed 3,081 genes with differential expression, including 1,310 up-regulated genes and 1,771 down-regulated genes in the adult stage (Figure 4). 504 genes were uniquely expressed in the adult stage and 505 genes were uniquely expressed in the egg stage. The nine most up-regulated genes in the adult library were annotated (Table S4), *i*.*e*., four egg formation genes (*yolk polypeptide 2* and three *vitellogenin* ) and a cuticle protein gene (*putative cuticle protein*). The nine most down-regulated genes included four cuticular protein genes (*cuticular protein CPG8*, *cuticular protein RR-1 motif 44*, *cuticular protein RR-2 motif 101* and *cuticular protein CPG7*) and an enzyme inhibitor gene (*serine protease inhibitor 8*). In the COG classification (Table S5) the up-regulated genes in the adult stage were involved in posttranslational modification, protein turnover, chaperones, energy production and conversion, nucleotide transport and metabolism and inorganic ion transport and metabolism.

There were 4,880 genes with demonstrated significant changes between the pupa and egg libraries (Figure 4). Among these genes, 1,958 were up-regulated and 2,922 were down-regulated in the pupa stage. 1,012 genes were uniquely expressed in the pupa and 826 genes were uniquely expressed in eggs. All ten of the most up-regulated genes in the pupa library have predicted functions (Table S4), *i*.*e*., two tonB genes (two *protein tonB, putative*), an enzyme gene (*similar to Chymotrypsin-2*) and a *beta-tubulin* gene. Eight of the ten most down-regulated genes were annotated, including four cuticular protein genes (*cuticular protein CPG8*, *cuticular protein RR-1 motif 44*, *cuticular protein RR-2 motif 101*, *cuticular protein CPG7*). According to the COG classification genes up-regulated in the pupa stage compared to the egg were less identifiable to function than genes down-regulated in the pupa stage, except for cell wall, membrane and envelope biogenesis.

## Discussion

High throughput transcriptome sequencing technology is a relatively new experimental technology that is continuously undergoing improvements. Next-gen sequencing has become an indispensable tool for genomics studies and has been widely used in a variety of important biological research. We compared the transcriptomes of four developmental stages of the maize pest *A.* *lepigone* to provide a framework for understanding changes in gene expression during development. We assembled a total of 81,356 unigenes with an average length of 612 bp and 12,070 unigenes longer than 1000 bp. The Illumina sequencing depth and assembly efficiency in this study are improvements over previous reports [16], [19].

The generated 81,356 unigenes were searched against the non-redundant (nr) NCBI protein database using BLASTX. A total of 33,736 unigenes were returned. The sequence matching results showed that *A.* *lepigone* shared the highest similarity (20.23% of the annotated unigenes) with the red flour beetle *T*. *castaneum* (Coleoptera) rather than the silkworm *B*. *mori* (9.62%) of the same order. The same pattern was found in a transcriptome study of the lepidopteran diamondback moth *Plutella xylostella* [23]. In addition, a similar pattern was also found in the transcriptome study of the back plant hopper and the white back plant hopper (WBPH) [16], [19]. These species exhibited a similarity of 18.89% and 16.17% matches with *T*. *castaneum* and 13.19% and 12.49% with the pea aphid *Acyrthosiphon pisum* of the same order, respectively. These results are likely due to the availability of more sequence resources of *T*. *castaneum* compared to *B*. *mori* and *A.* *pisum* in the NCBI protein database, as the numbers of protein sequences of *T. castaneum*, *B*. *mori* and *A.* *pisum* in NCBI database were 27316, 7848 and 20463, respectively.

Fast-evolving molecular markers are important tools for the study of population genetics and we identified 2,819 microsatellites in the transcriptome of *A.* *lepigone*. Our results indicate that the most common trinucleotide repeats in *A.* *lepigone* are similar to *Sogatella furcifera* and *Laodelphax striatellus*, in that they have a frequent motif of (AAG)_n_ [16], [24]. The most common dinucleotide repeats GC/TG/AT/TA that we found in *A.* *lepigone* have not been described in other reports. For example, AG/GA/CT/TC motif repeats are more frequently found in *S. furcifera* [16] and there is no (GC)_n_ motif found in *L. striatellus* [24]. Our results indicate that the dinucleotide SSRs recovered here are distinctive to *A.* *lepigone*, although data on dinucleotide SSR variability is still limited for most insect orders. The (GA)_n_ motif is known to regulate gene expression in animals and plants [25]–[27], but only a few (GA)_n_ motifs exist in *A.* *lepigone*. Further study is needed to test whether these (GA)_n_ motif repeats retain a regulatory function in *A.* *lepigone* or if the function of (GA)_n_ has been replaced by other motif repeats. Moreover, the large numbers of potential molecular markers found in our study will be particularly useful for future genetic mapping, parentage analysis, genotyping and gene flow studies of this species.

Many differentially expressed genes were obtained by making pair-wise comparisons of the transcriptomes of four developmental stages. During the development of *A.* *lepigone* most of the differentially expressed genes were down-regulated in the larva stage compared to egg stage, and most of genes were up-regulated in the pupa stage compared to larva stage. The genes with the strongest difference in expression correlate with some of the characteristic features in the various development stages, for example, egg formation and flight protein related genes are highly expressed in the adult stage and correspond with some of the key life activities of the adult.

Giving insights into ten of the most differentially up-regulated and ten of the most differentially down-regulated genes, most of these genes had predicted functions. Cuticular protein genes existed in the twenty most differentially expressed genes in the comparison between each library, except between the adult and pupa stages. This indicates that insect cuticular protein composition is expressed differently in different developmental stages. Insect cuticle is a compound of cuticular protein and chitin and not only supports and maintains the physical structure of the organism, but also serves as a natural barrier against external adverse impacts [28]. A change of cuticular protein determines the cuticle composition and performance and is the basis for explaining the performance of cuticle-based structures [29]. This result provides useful information for us to conduct further studies on the cuticular protein and cuticle.

Our study constructed four transcriptome libraries and compared the gene expression abundance of *A.* *lepigone* among different developmental stage. The annotated unigenes and unigene expression abundance provide useful information for the identification of genes involved in *A.* *lepigone* development.

## Supporting Information

Table S1 Top hits obtained by BLASTX for the unigenes. Note: BLASTX against the nr protein database was used with a cutoff E value <1e^−5^.
(XLS)

Table S2 GO annotation of unigenes.
(XLS)

Table S3 KEGG annotation of unigenes.
(XLS)

Table S4 Top ten differentially expressed genes in each library of comparisons. Note: The expression fold changes were performed with log 2 ratio.
(XLS)

Table S5 Gene set enrichment analysis comparing between each sample.
(XLS)
